# What Is Stressful About Medical Liability? A Retrospective Jurisprudential Analysis of Moral Damages and Discussions on Its Impact on Healthcare Professionals’ Well-Being

**DOI:** 10.3390/healthcare14172742

**Published:** 2026-08-28

**Authors:** Paula Roxana Adi, Bianca Hanganu, Rareș Dorin Pop, Camelia Liana Buhaș, Karla Croitorescu, Mircea Enache, Beatrice Gabriela Ioan

**Affiliations:** 1 Grigore T. Popa University of Medicine and Pharmacy Iasi, 700115 Iasi, Romania; adipaularoxana@gmail.com (P.R.A.); beatrice.ioan@umfiasi.ro (B.G.I.); 2 Dr. Constantin Opriș Emergency Clinical Hospital, 430031 Baia Mare, Romania; rarespop75@gmail.com; 3 Department of Morphological Disciplines, Faculty of Medicine and Pharmacy, University of Oradea, 410087 Oradea, Romania; cameliabuhas@yahoo.com; 4 Faculty of Medicine, George Emil Palade University of Medicine, Pharmacy, Science and Technology of Târgu Mureș, 540142 Târgu Mureș, Romania; karla.2003.croit@gmail.com; 5 Mureș County Clinical Hospital, 540072 Târgu Mureș, Romania; enache_mircea320@yahoo.com

**Keywords:** moral damages, medical liability, judicial predictability, healthcare professionals, well-being, mental health, litigation stress, Romania

## Abstract

**Background/Objectives:** Medical liability litigation in Romania has risen steadily over the past decade, yet the quantification of moral (non-pecuniary) damages remains entirely at the discretion of each judge, with no standardized reference. This raises concerns about consistency and predictability, with direct implications for healthcare professionals, who are increasingly exposed to this form of liability—a potential source of occupational stress—and to financial awards they cannot anticipate. The present pilot study analyzed Romanian court practice regarding the amount of moral damages awarded in cases of medical negligence resulting in the patient’s death. **Methods:** In a retrospective observational study, we examined ten final rulings (2018–2024; rejust.ro) convicting medical personnel for involuntary manslaughter (27 civil parties awarded). We computed the Claim-to-Award Ratio (CTAR), Claim Reduction Rate (CRR), Appeal Adjustment Rate (AAR), and Appeal Ratio (AR), Spearman correlation, the Wilcoxon signed-rank test, and linear regression. **Results:** Courts awarded on average only 18% of the amounts claimed (CRR 82%); claimed and awarded sums were correlated (ρ = 0.84, 95% CI 0.34–0.97, *p* = 0.0025; R^2^ = 0.59). Appellate adjustments varied widely (AAR −84% to +79%; AR 0.16–1.79), with no systematic difference between instances (Wilcoxon *p* = 0.463; R^2^ = 0.01). Comparable cases yielded substantially different awards. **Conclusions:** Moral damages in fatal malpractice show no recognizable pattern of quantification. This structural unpredictability leaves physicians without a reliable benchmark for their financial risk and may amplify the occupational stress and impaired well-being already linked to litigation, supporting the need for guiding reference standards.

## 1. Introduction

Legal liability represents the coercive expression of the authority of the rule-of-law state, activated whenever the legal order is breached by an unlawful act, and its role is to restore legality by applying the consequences provided by the rules of law against the liable person [1]. In the case of medical personnel, legal liability may take distinct forms, depending on the nature of the breached obligation and the gravity of the resulting consequences, namely disciplinary, civil, and criminal liability [2]. Thus, conduct that does not conform to the standard of practice may give rise to professional sanctions for the breach of ethical norms and service duties, the obligation to repair the harm caused to the patient or, in cases of particular gravity, criminal liability for acts provided by criminal law [3].

In Romania, malpractice is defined as “the professional error committed in the exercise of the medical act, generating harm to the patient, entailing the civil liability of the medical personnel” [4]. Thus, medical personnel who provide medical services are liable for the harm caused by error (consisting of negligence, imprudence, or insufficient knowledge), the exceeding of competences, and the failure to observe the regulations concerning confidentiality, consent, and the obligation to provide medical assistance [4]. Moreover, where the unlawful conduct meets the constitutive elements of an offense as provided by criminal law (namely that it is committed with guilt, is unjustified, and is imputable to the person who committed it) [5,6], medical personnel may also be held criminally liable, independently of or concurrently with the other forms of legal liability.

The harm resulting from a medical act may vary in forms and degrees of severity, ranging from violation of patients’ rights, minor complications and a prolongation of the recovery period, to permanent disability or even the patient’s death.

Under the rules of civil liability, the patient or, as the case may be, their relatives, may seek the reparation of the harm suffered, having the possibility of obtaining compensation for the consequences produced by the unlawful medical act. These consequences may take two forms: a material (pecuniary) one and a moral (non-pecuniary) one.

Pecuniary harm consists of the reduction in the value of a person’s patrimony, as a result of the material losses generated, namely the loss actually suffered (*damnum emergens*) such as hospitalization and recovery costs, the purchase of medicines or medical devices, expenses for permanent care and assistance, and medical transportation expenses, as well as funeral expenses in the event of the patient’s death [7,8].

Non-pecuniary harm refers to “the restriction of the possibilities of family and social life” or, as the case may be, to “the pain experienced through the death of the victim” (*pretium doloris*) and may be repaired through the court’s provisions, that is awarding compensation for the non-pecuniary damages caused to the patient, ascendants, descendants, brothers, sisters, and the spouse, as well as to any other person who, in turn, could prove the existence of such harm [8,9]. The award of non-pecuniary damages in courts is based on a principle according to which “a physical pain or a psychological suffering may be alleviated, to a greater or lesser extent, (…), through the realization of a satisfaction”, more precisely of monetary compensation [10].

The interest in the issue of medical liability is justified by the increasing magnitude of medical litigation in Romania, reflected in the steadily rising number of medical malpractice claims and their expanding professional and social impact. The analysis carried out in 2018 by Dumitrescu highlights a significant upward trend in litigation concerning medical malpractice in Romania, the number of identified cases recording an increase of more than eight-fold over the past 11 years [11]. Recent studies show that the risk of being accused of medical malpractice in Romania is approximately 16% among physicians [12].

Moreover, according to the statistical data communicated by the Superior Council of Magistracy, more than 50% of the medical personnel who reach the criminal court are convicted and ordered to pay material and non-pecuniary damages [13].

Material (pecuniary) damages may be awarded only when the pecuniary harm is proven, both as to its existence and its extent, by supporting documents or other evidence capable of demonstrating the actual loss suffered. On the other hand, in the practice of the Romanian courts, the judge, through their professional independence [14], establishes in each case the amount of the non-pecuniary damages, without being constrained or influenced [7]. This freedom allows, on the one hand, the individualization of the pronounced solution in accordance with the particularities of the case and professional experience, but, at the same time, it raises the problem of delimiting common benchmarks that would allow a uniform practice at the national level.

By comparison, the Spanish system relies on the standardization of harm assessment, through the use of the Baremo table, a system for quantifying compensation for bodily harm, updated annually through the publication of the new indemnity amounts. Although conceived for the matter of traffic accidents, it is used in practice as a benchmark in cases concerning medical legal liability, precisely due to its capacity to introduce objectifiable criteria in the assessment of harm [15]. Baremo is the only system for quantifying non-pecuniary damages in Europe that distinguishes between the harm resulting from death, permanent disability, and temporary injuries, integrating criteria for individualizing the amount of compensation such as the age of the victim, the family relationship with the harmed persons, economic dependence, lost income, medical and funeral expenses, as well as the personal and family impact of the harmful event. In this way, the Spanish model does not eliminate the role of the court in individualizing compensation, but it offers quantifiable benchmarks, making outcomes more predictable and reducing variation between rulings [16,17].

Both in Romania and at the international level, the analysis of the quantification of non-pecuniary damages has been explored predominantly in the matter of compulsory motor third-party liability insurance (RCA), a field in which guidelines, guiding benchmarks, and trauma points for bodily harm have already been developed [7,18,19]. Reference instruments such as the Ogden Tables in the United Kingdom, the Baremo System in Spain, or the Mornet Reference Framework in France were natively structured as general mechanisms and guidelines for the compensation of bodily harm applicable in courts [16,17,20,21].

By contrast, medical negligence has remained unexplored from an econometric and statistical perspective, the analysis of the literature usually being limited to theoretical approaches to medical liability or to general statistics concerning the incidence of complaints. The absence of an explicit methodological framework for assessing non-pecuniary damages in medical malpractice cases may result in significant disparities in the amount awarded. Unlike the RCA system, in which established benchmarks attenuate the jurisprudential gap, in medical negligence the dependence on the individual appreciation of the panel of judges is absolute.

This centrality of the courts is reinforced by the way in which compensation for medical harm is actually pursued in Romania. Theoretically, claims for material and moral damages may be settled out of court through mediation [22]; however, practical implementation of mediation is limited [23], so that the vast majority of cases reach the courts. Litigation may take place before a civil court or, where the conduct also constitutes a criminal offense, before a criminal court; in the latter case the criminal court may either sever the civil action—the claim for reparation of the harm—and refer it to a civil panel, or adjudicate it itself [24]. Pre-trial settlement is not an established practice in Romania, and there is no functioning mechanism whereby a hospital committee assesses claims and proposes a settlement before litigation; virtually all monetary claims (material and, above all, moral) are therefore quantified and resolved by the courts. Any administrative avenues that may precede litigation do not quantify the damages themselves, and their outcomes are in any event routinely contested before the courts, so the amount of the award is ultimately determined judicially.

Beyond its strictly legal dimension, medical malpractice litigation is a significant occupational stressor for healthcare professionals. Being named in a liability claim—particularly a criminal one—has been described as a traumatic experience in its own right, and associated with anxiety, depression, burnout, and defensive practice [25,26,27,28]. In Romania, where the number of malpractice actions has grown steadily, physicians and nurses are therefore exposed not only to the legal and financial consequences of liability but also to its psychological burden.

The aim of the present study is to analyze the practice of the Romanian courts concerning the extent of the amount of non-pecuniary damages awarded in cases of medical negligence resulting in the death of the patient, through a descriptive and statistical evaluation of the values established in jurisprudence. This aim is pursued along three converging analytical axes: (i) the amounts claimed vs. those awarded; (ii) the amounts established at first instance vs. on appeal; and (iii) the amounts awarded in factually comparable cases. Beyond this descriptive aim, the study further discusses the potential implications of the observed unpredictability for the occupational stress and well-being of the physicians and nurses exposed to such litigation.

## 2. Materials and Methods

The present study has a retrospective observational design, based on the analysis of court rulings pronounced in Romania over a period of 6 years (2018–2024), in cases of medical negligence resulting in the death of the patient.

The temporal delimitation of the sample, having as its starting point the year 2018, is grounded in the dynamics of litigation reported in the specialized literature [11]. The reference study carried out by Dumitrescu retrospectively analyzed the malpractice files in Romania registered up to 1 April 2018, highlighting an exponential increase in the litigation phenomenon and forecasting a historical peak of accusations precisely for the course of that year. The present study ensures the continuity of the national jurisprudential analysis and allows the evaluation of the way in which the quantitative explosion in the number of proceedings signaled by the author was reflected qualitatively and financially in the decisions of the first-instance and appellate courts.

### 2.1. Data Collection

The cases were identified by accessing the rejust.ro portal, an application developed by the Superior Council of Magistracy of Romania, which facilitates the access of citizens and jurists to the rulings pronounced by the national courts. The portal includes court rulings and procedural conclusions, in anonymized format, originating from all courts. Within the portal, advanced search filters were applied in order to identify the cases that complied with the following inclusion criteria for the study: rulings pronounced in the period 1 January 2018–31 December 2024, in district courts and tribunals in Romania, having as their legal matter the criminal aspect, classified as “negligent homicide” provided for and punished by the Romanian Criminal Code at art. 192 para. (2), in which the pronounced solution was the conviction of the medical personnel. This filtered search returned 3535 rulings. Within this set we searched using the combinations of relevant keywords such as “physician”, “malpractice”, “patient”, “medical negligence”. Each returned ruling provided by the platform was analyzed manually, reading the “court’s reasoning” section, where the facts are described, in order to exclude those that did not meet the inclusion criteria, such as those unrelated to the medical act and the cases in which the solution was acquittal. This manual screening yielded the final sample of ten convictions. These ten convictions represent the complete set of eligible cases (final criminal convictions for medical negligence resulting in the death of the patient) identified in the national jurisprudence for the entire 2018–2024 period; the small number therefore reflects the genuine rarity of such convictions rather than a sampling decision.

For each case, the following variables were extracted: (1) the category of the entitled person (husband/wife, child, parent, etc.); (2) the amount of non-pecuniary damages claimed by each civil party; (3) the amount of non-pecuniary damages awarded at first instance for each civil party; (4) the amount of non-pecuniary damages awarded on appeal for each civil party; and (5) the total value of non-pecuniary compensation awarded per case (all civil parties).

### 2.2. Data Processing

To facilitate an objective and standardized analysis of the judicial assessment of non-pecuniary damages, the raw financial data were subjected to a stage of mathematical processing. Four original proportional indicators were developed to standardize the asymmetric raw data, minimize differences in scale, and facilitate the identification of underlying patterns in judicial practice. What we sought from these indicators was to extract the decision-making pattern of the courts and to respond in a standardized manner to a set of questions concerning the mechanisms of the legal reparation of non-pecuniary harm. Thus, for each case included in the sample, the following key indicators were defined and calculated, as appropriate:

- Claim-to-Award Ratio (CTAR), which measures the degree of coverage of the amounts claimed in the pronounced rulings (clarifying what percentage of what was claimed was ultimately awarded);
- Claim Reduction Rate (CRR), which tracks the intensity of the reduction applied by the courts to the amounts claimed;
- AAR (Appeal Adjustment Rate), which captures, in percentage terms, the intervention of the appellate court on the amounts established by the first-instance court;
- AR (Appeal Ratio), which acts as a direct multiplier showing how many times the value of the final compensation oscillated relative to that of the first instance.

The four indicators were calculated in order to allow us, ultimately, an objective evaluation of the concepts of equity and predictability in the matter of medical liability.

For the calculation of these indicators, we considered the moral damages claimed (RD—requested damages) and those awarded (AD—awarded damages), as well as the amounts established at first instance (FI—first instance) and on appeal (AC—appellate court). The choice of these four indicators follows directly from the two questions the study asks: because the monetary amounts differ by orders of magnitude across cases and cannot be compared directly, the indicators are dimensionless ratios that normalize them. CTAR and its complement CRR express the claim-to-award relationship, whereas AR and AAR express the first-instance-to-appeal relationship; within each pair the two indicators are two expressions of the same quantity, and none is privileged, the four being complementary and jointly characterizing the two dimensions of predictability examined here. The four indicators were thus computed using the following formulas:

(1) $$CTAR=\frac{AD}{RD}$$

CRR = 1 − CTAR(2)

(3) $$AAR=\frac{AC-FI}{FI}$$

(4) $$AR=\frac{AC}{FI}$$

All ten convictions were included, whether the case concluded at first instance or on appeal. The CTAR and CRR indicators, which capture the relationship between the amount claimed and the amount awarded, were calculated for all ten cases using the final awarded amount. In contrast, the AAR and AR indicators, which measure the adjustment made by the appellate court, were calculated only for the analyzed cases that reached the appeal, and subsequently their average was then determined for this appellate subsample of cases.

### 2.3. Statistical Analysis

The extracted data and the calculated indicators were subjected to descriptive and inferential processing. Descriptive statistics targeted the distribution of frequencies, the establishment of value ranges, and the determination of the arithmetic means at the level of the sample and subsample selected for each type of analysis. Inferential statistics were structured on specific tests described below. The calculation of the Spearman correlation coefficient and the application of the Wilcoxon signed-rank test were carried out using the digital statistical platform Social Science Statistics (https://www.socscistatistics.com accessed on 6 March 2026). The primary processing of the raw data, the calculation of the mathematical indicators, and the modeling of the simple linear regressions (together with the generation of the related graphical representations) were carried out using the statistical functions of the program Excel Microsoft Office (version 16.112.1). The 95% confidence interval for Spearman’s ρ was computed by the Fisher z-transformation using the Bonett–Wright standard error. Given the small sample, the statistical analyses are exploratory and are interpreted as hypothesis-generating.

In order to evaluate the correlation between RD per case and AD per case, we used the Spearman coefficient (ρ). The choice of the statistical test was determined by the small volume of data and by the presence of extreme values among the entered data (e.g., claims amounting to thousands or even millions of euros). Statistical significance was evaluated by testing the null hypothesis, represented by the absence of association between RD and AD, using a significance threshold of *p* = 0.05 (two-tailed *p*).

To evaluate the existence of a systematic difference between the amount of non-pecuniary damages awarded by FI and established by AC, we used the Wilcoxon signed-rank test for paired samples, given the limited volume of the data and their paired character, each case being evaluated at two distinct jurisdictional moments: the first instance (FI) and the appellate court (AC). The Wilcoxon test analyzes the distribution of the differences between the paired values and allows us to evaluate the hypothesis according to which there is no systematic difference between the amount of compensation established by FI and that established in AC (with a level of statistical significance set at α = 0.05).

To explore the relationship between RD and AD, as well as FI and AC, a simple linear regression analysis was also carried out, using RD and FI as independent variables and AD and AC as dependent variables.

The results were represented graphically through a scatter plot, in which each point represents an analyzed case. The graph included both the linear regression line and the theoretical line of equality (y = x).

The degree of association between the two variables was evaluated through the coefficient of determination (R^2^), which expresses the proportion of the variation in AD explained by the variation in RD, and the proportion of AC was explained by the variation in FI.

### 2.4. Qualitative Analysis

The court rulings included in the sample (*n* = 10) were examined individually, through full reading, in order to determine the factual circumstances of each case. The analysis aimed at identifying situations in which the relevant elements of the non-pecuniary harm presented significant similarities, such as the type of family relationship between the deceased person and the civil party, the age of the persons involved or of the civil parties. The purpose of this analysis was to highlight the way in which the courts established the amount of non-pecuniary compensation in comparable situations, seeking to identify any variations thereof. Specifically, for each ruling, we extracted, from the “court’s reasoning” section, the identity and role of the deceased, the family relationship of each civil party to the deceased, and the age of the persons involved; on this basis, factually comparable cases were grouped and the amounts awarded within each group were compared.

The qualitative evaluation of the sample allowed the classification of the cases into categories of cases, in which the amounts of non-pecuniary compensation awarded were analyzed through simple mathematical comparison, seeking the variability of the raw values, both at the level of the first instance and, where applicable, at the level of the appellate court.

## 3. Results

As described in Section 2.1, the authors analyzed the ten final convictions identified (physicians and nurses convicted of negligent homicide associated with deficient medical care). Within these, the non-pecuniary compensation claimed by a number of 28 civil parties and awarded to a number of 27 persons (one was removed from the award of compensation because the civil parties had died) was evaluated, represented by life partners (husband/wife) and first-degree relatives (sons, daughters, parents) and second-degree relatives (brothers, sisters). Of all the claiming civil parties, 5 were husbands/wives, 6 were children, 11 were parents, and 6 were brothers/sisters.

The highest civil claims amounted to €1,500,000 per party, respectively €4,000,000 per case, while the lowest were €4000 per party and €22,000 per case. In total, summing the claims from the ten cases, claims for non-pecuniary damages amounting to €13,172,000 were submitted. In two of the ten cases, the claimed compensation was differentiated based on the civil party’s relationship to the deceased. In one case, the parents claimed €4000 and the siblings claimed €4000; in the second case, the parents claimed €200,000 and the siblings claimed €100,000.

The total amount of non-pecuniary compensation awarded per case varied between €17,000 and €400,000, with a total of €1,147,000 for the ten studied cases. The average of the non-pecuniary damages awarded was approximately €42,481 per civil party (Table 1).

The damages awarded by the first-instance court started at €3000 per civil party and increased approximately 83-fold, reaching a maximum of €250,000 awarded to a single civil party. In six of the ten cases, the first-instance rulings were modified on appeal with respect to the amount; thus, the appellate court reduced the compensation per civil party the most by €210,000 (6.25 times compared to the first instance) and increased it the most by €117,000 (2.4 times). In 50% of the retried cases, the non-pecuniary damages were diminished by the appellate court, by at least €25,000 per party, while in the remaining cases the court increased the damages by €5000, €20,000, €25,000, and €117,000 (with differences according to the capacity of the civil party).

The smallest difference between the damages claimed and those awarded per case in the court’s final ruling was €5000, while the largest was a decrease of €3,600,000 between the amount claimed and those established by the appellate court.

**Figure 1:**
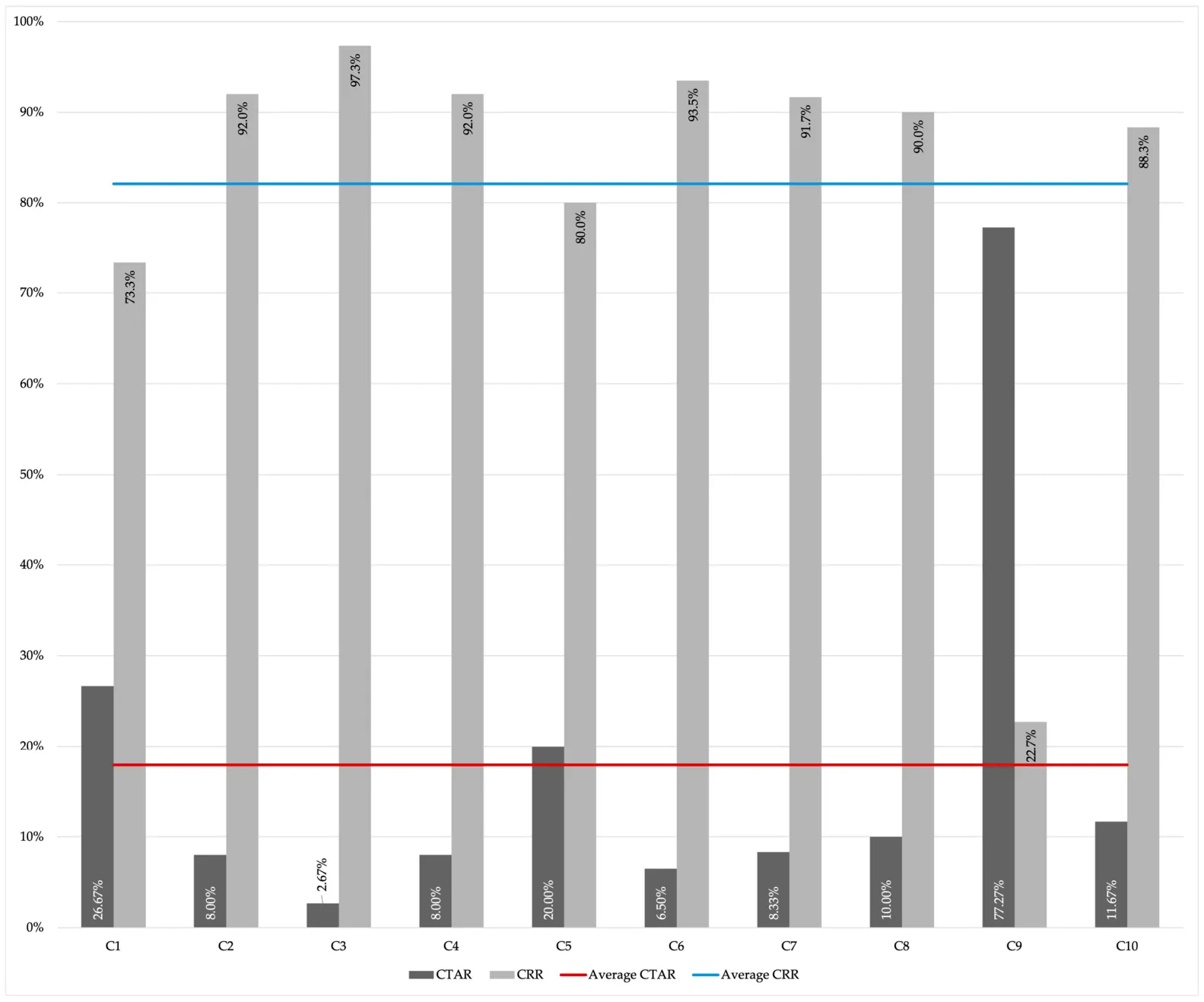
Representation of CTAR and CRR for each case and their average.

The average value of the CTAR indicator was 0.179 (18%), corresponding to an average CRR of 82% (Figure 1). The individual CTAR values varied between cases, from 2.67% up to 77.27% in a case in which the difference between the amount claimed and the amount awarded was €5000.

The Spearman correlation analysis between RD and AD had a statistically significant result, *n* = 10, ρ = 0.84, *p* = 0.0025, 95% CI 0.34–0.97.

The linear regression analysis highlighted the existence of a positive relationship between RD and AD, below the line y = x, described by the following equation:

(5) y=0.0688x+24034

**Figure 2:**
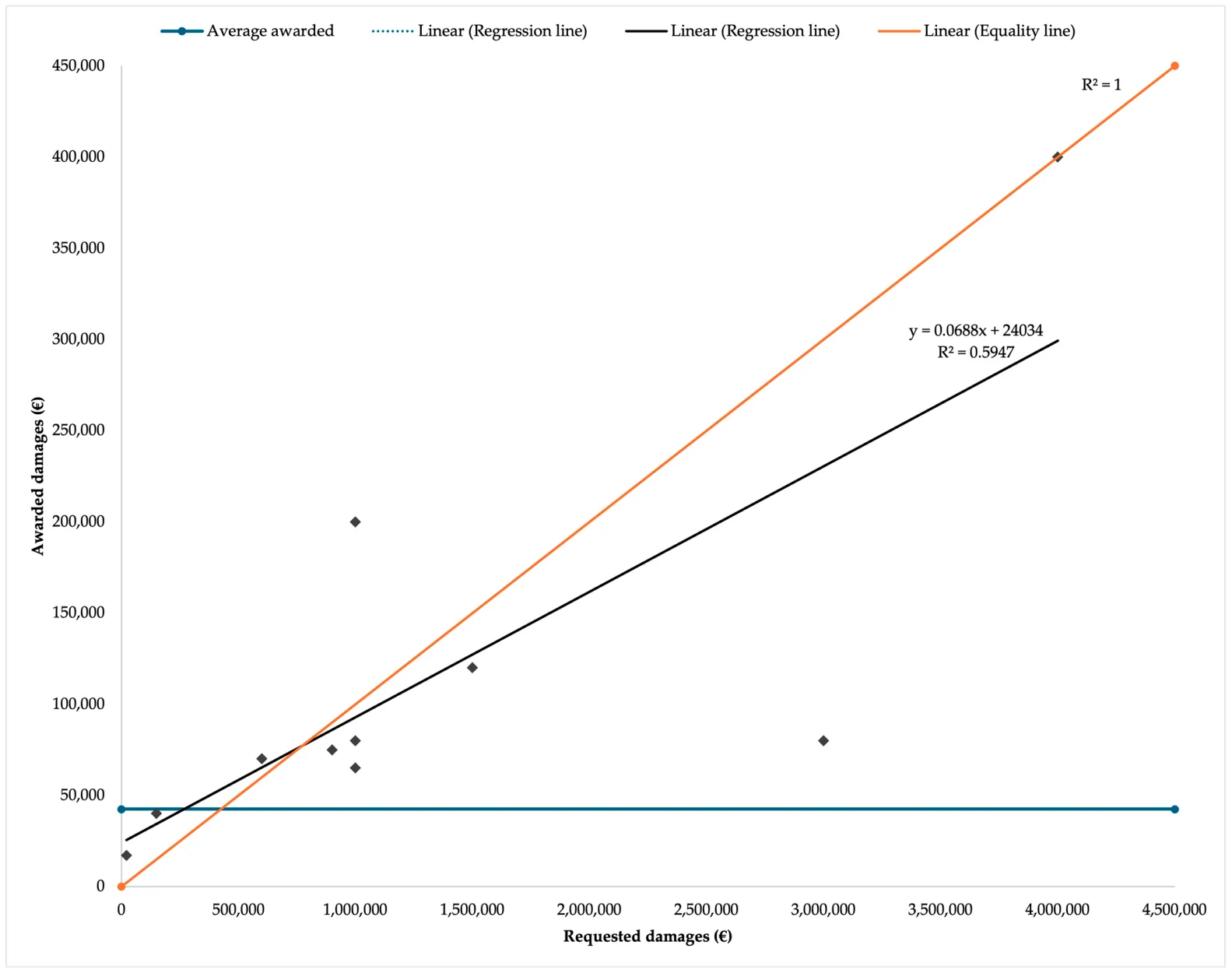
Regression analysis for each case, the line of equality and average awarded.

The coefficient of determination obtained (R^2^ = 0.5947) indicates that approximately 59.47% of the variation in AD can be explained by the variation in RD (Figure 2).

**Figure 3:**
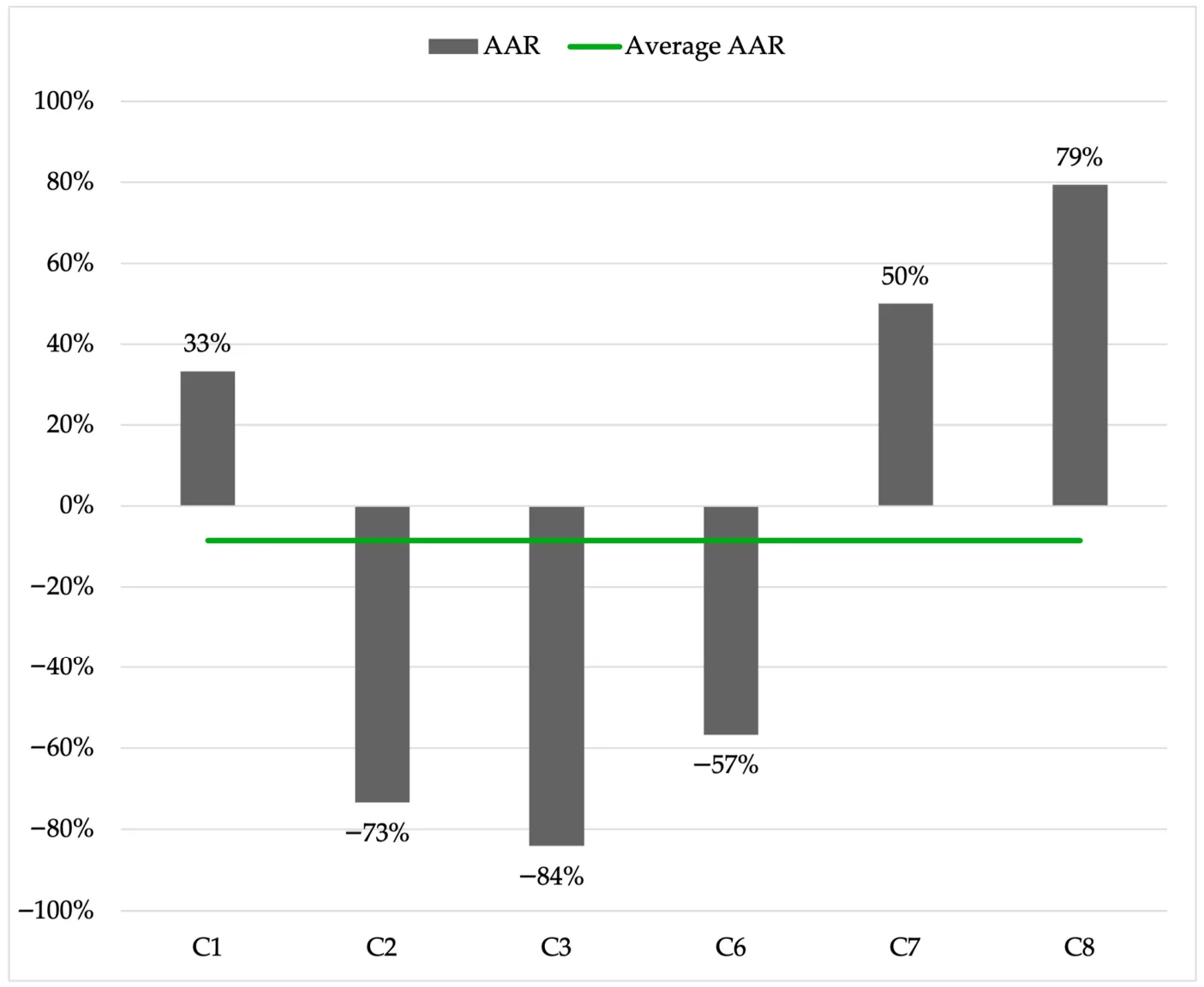
Representation of AAR for each case and the average.

The AAR values varied between −84% and +79%; in three of the analyzed cases, AC ordered the increase in the non-pecuniary compensation, with increases of 33%, 50%, and 79%, while in the other three cases the amount established by FI was reduced, with decreases of 57%, 73%, and 84%. The average of the AAR values for the entire subsample was approximately −9% (−8.7%), under conditions of high variability between cases (Figure 3).

The AR indicator recorded values between 0.16 and 1.79. AR values smaller than 1 indicated the situations in which AC reduced the compensation established by FI, while values greater than 1 reflected the cases in which it was increased (Table 2).

In the analyzed cases, the appellate court awarded between 16% and 43% of the amount initially established by the first instance in the situations of reduction, respectively between 133% and 179% of the initial amount in the cases of increase.

The application of the Wilcoxon signed-rank test revealed that there is insufficient statistical evidence to support the existence of a systematic difference between the amount of non-pecuniary damages awarded by FI and the amount established by AC (w = 7, Z = −0.7338, *p* = 0.463).

**Figure 4:**
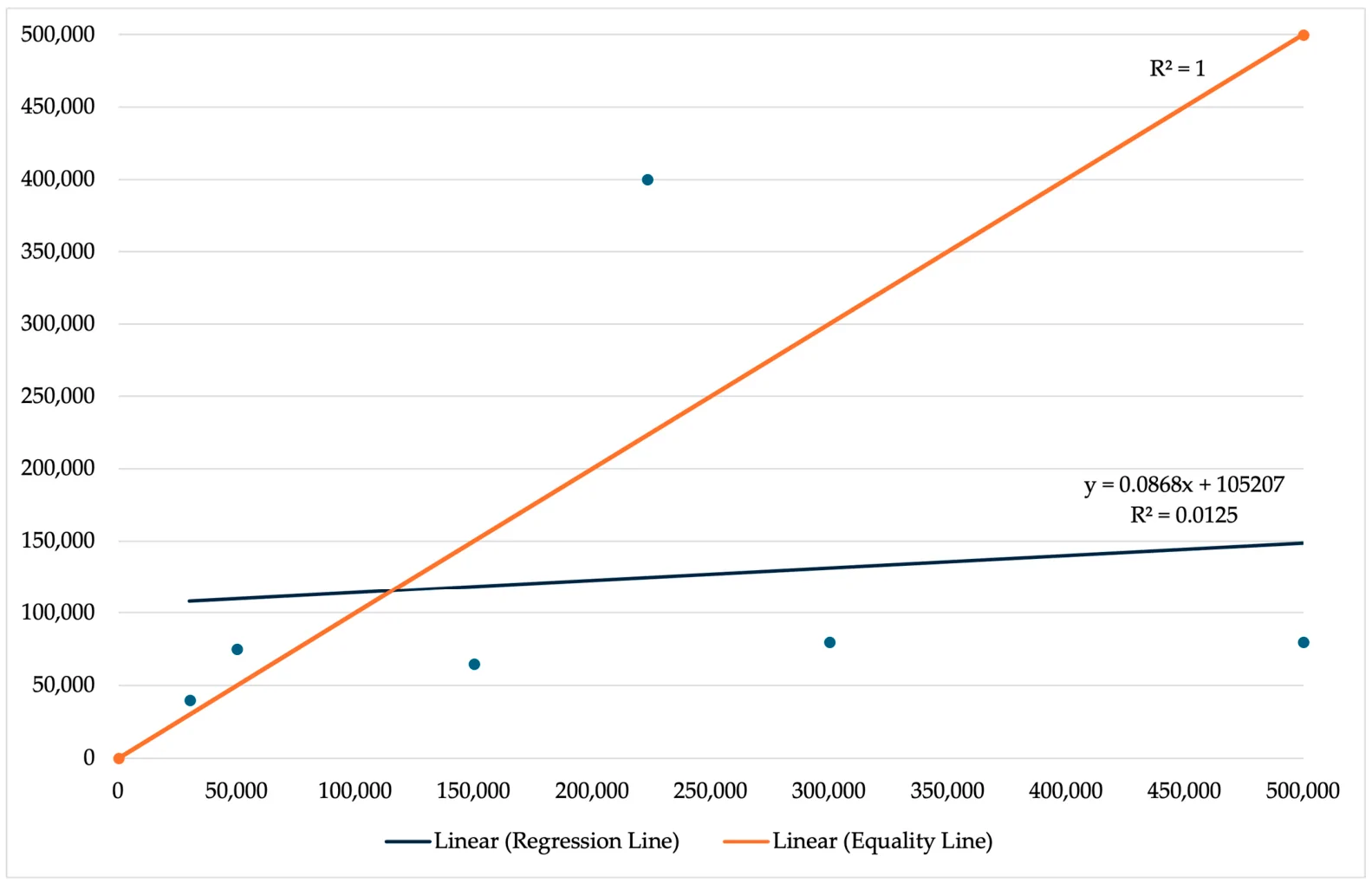
Regression analysis for each case and the line of equality.

The linear regression analysis carried out between FI and AC highlighted a very weak relationship between the two variables (Figure 4). The coefficient of determination was R^2^ = 0.0125, and the resulting regression equation was

(6) y=0.0868x+105207

This result indicates that only approximately 1.25% of the variation in the amount of compensation awarded on appeal can be explained by the amount established by the first instance, suggesting the absence of a relevant linear relationship between the two jurisdictional levels.

Following the qualitative analysis of the court rulings (*n* = 10), three categories of comparable cases were identified:

(1) Cases concerning the death of newborns, in which the civil parties were the parents (Table 3);
(2) Cases concerning the death of a wife (and mother), in which the civil parties were close family members such as husband, child, parents (Table 4);
(3) Cases in which the civil party was a minor child left without one of the parents (Table 5).

In three of the analyzed cases, the death concerned newborns. In two of these, the civil parties were exclusively the parents, and in the third case the victim’s brother was also constituted as a civil party. The amount of compensation claimed was €100,000 per civil party (the lowest per case being €500,000) and 15 times higher in one of the cases where €1,500,000 was claimed for each civil party.

At first instance, the compensation awarded per case varied between €75,000 and €250,000, being differentiated according to the capacity of the civil party in one of the cases. In this case, the smallest amount awarded was €5000, approximately 25 times smaller than the largest compensation awarded to a single person within the same set of cases. In two of the three cases, the amount of compensation was modified on appeal, the court establishing the sum of €40,000 for each civil party.

Two of the cases concerned the death of an adult female person, in both situations the victim’s husband being constituted as a civil party, and in one of the cases also the adult son and the parents of the deceased. The sums claimed were €900,000 and €1,000,000 per case. At first instance, the amount awarded to the husband was €50,000 in one case and €83,000 in the other. On appeal, these sums were increased by €25,000 and €117,000 respectively. In the case in which there were several civil parties, the compensation was differentiated between husband, son, and parents, with differences of up to €150,000 between the amounts awarded to the civil parties.

In two of the analyzed cases, the civil parties were minor children of similar ages, of persons who died at a similar age. In the first case, a child was awarded compensation of €80,000 at first instance, a sum increased on appeal to €100,000. In the second case, a child was awarded compensation of €5000, the first-instance ruling remaining final.

## 4. Discussion

### 4.1. Amount Claimed vs. Amount Awarded

The comparison between the amounts claimed (RD) and those awarded (AD) in these fatal-negligence cases highlights several relevant tendencies in how the courts set non-pecuniary compensation relative to the claims.

The analysis indicates that the courts award, on average, approximately 18% of the amount of non-pecuniary compensation claimed, corresponding to an average reduction rate of 82%. The result suggests the existence of a significant difference between the level of the claims formulated by the civil parties and the amount of compensation considered equitable by the court.

The positive correlation between RD and AD (ρ = 0.84, *p* < 0.003) suggests that the amounts claimed are associated with the compensation awarded, and that approximately 59% of the variation in AD can be explained by the variation in RD (R^2^ = 0.5947). In other words, the cases in which larger compensation is claimed tend to conclude, on average, with larger compensation awarded. Moreover, the study shows that for each additional monetary unit claimed, the compensation awarded increases by approximately 0.06 monetary units (regression coefficient = 0.0688). For example, if RD increases by €100,000, the court will increase AD on average by approximately €6000. The intercept of this regression (≈ €24,034) can be read as a baseline award largely independent of the amount claimed: the courts appear to grant all claimants a floor and then to scale the award only modestly according to the sum requested.

This interdependence was also observed as a tendency in the study by Campbell et al. [29], based on simulations of medical malpractice trials, which showed that the AD value is profoundly influenced by the RD amount due to the anchoring phenomenon (anchoring effect). Kahneman and Tversky [30] described anchoring as a cognitive bias whereby the estimation of an uncertain value is strongly drawn toward a number provided as an initial reference point, with the brain failing to sufficiently adjust its subsequent estimates relative to this mental anchor. Applied to the dynamics of Romanian courts, this psychological determinism may help explain why, despite the substantial average reduction rate identified in our sample (CRR = 82%), the courts appeared to remain influenced by the level claimed by the civil parties (RD). It should nonetheless be acknowledged that this association admits a second, non-exclusive interpretation: rather than a cognitive anchoring effect, the correlation may reflect that factually worse cases attract both higher claims and higher awards, so that it tracks the severity of the harm. Distinguishing these explanations requires systematic coding of severity-related features; while the age of the victim was recorded for the present research (and underpins the grouping of comparable cases), other relevant features such as the clinical circumstances and the degree of fault are present in the rulings but were not extracted in this pilot study. We therefore treat the anchoring interpretation as a hypothesis to be tested rather than a demonstrated mechanism, and we identify its verification, through larger samples and a more granular, segment-by-segment coding of the judicial reasoning, as a specific objective for future research. Our study aligns with the findings observed by the previously cited authors, validating the persistence of this cognitive distortion in the act of justice, where higher claims shift the quantification decision toward higher sums [31,32].

Nevertheless, the low slope of the regression suggests that this relationship is limited from a proportional point of view, the courts systematically awarding only a fraction of the amounts claimed. The graphical distribution of the regression (Figure 2) indicates that AD was smaller than RD (representation below the theoretical line of equality x = y) in all the analyzed cases.

Interpreted together, these results suggest that the process of establishing the amount of non-pecuniary damages involves two apparently complementary mechanisms. On the one hand, the courts appear to take into account the level of the compensation claimed, and on the other hand, the courts exercise a substantial reduction in these sums, establishing compensation significantly smaller than that claimed.

Taken together with the first-instance vs. appeal comparison (Section 4.2) and the differences between factually comparable cases (Section 4.3), which will be presented further, this gap between claimed and awarded amounts forms the first of the study’s converging observations of unpredictability, which jointly ground the paper’s central conclusion.

### 4.2. Amount Established at First Instance vs. On Appeal

The comparison between the amount of non-pecuniary damages established by FI and those awarded in AC shows considerable variability in the intervention of the higher court in the rulings pronounced by the lower court. The indicators used (AAR and AR) allowed the quantification of the extent of the modifications operated by the appellate court, highlighting the fact that these may lead to significant changes in the compensation awarded.

AAR varied within a wide interval, (−84%; +79%), and AR oscillated between 0.16 and 1.79, which reflects situations in which the appellate court awarded only a fraction of the sum initially established by the trial court, but also cases in which it increased the compensation by up to almost double the initial amount; in both perspectives, the intervention of the appellate court is substantial. The amplitude of the adjustments suggests that the appellate court does not function exclusively as a mechanism of minor correction of the amounts established at first instance, but may carry out a genuine re-evaluation of the non-pecuniary damages for each case.

Nevertheless, the study indicates that AC does not show a consistent tendency to either increase or reduce the non-pecuniary damages established by FI (w = 7, Z = −0.7338, *p* = 0.463), and that the damages awarded at first instance account for only a very small proportion of the variation in the amount awarded on appeal (R^2^ = 0.0125). In other words, within this small subset of six appealed cases, the level of compensation initially established was not a meaningful predictor of the final value established by the appellate court.

The amplitude of the modifications from one jurisdictional level to another is also confirmed in the conclusions of Avraham and Bustos [33], who demonstrate that, when the rules for establishing non-pecuniary damages are perceived as uncertain or liable to be overturned at a higher level, the behavior of the parties in the trial changes radically. They showed that the initial ruling does not function as a reference point, but rather the subjects anticipate a re-evaluation on appeal and choose to prolong the litigation in hope of obtaining higher amounts. Our indicators (AAR and AR) directly validate the existence of this mechanism in Romanian practice, demonstrating that the appellate court does not carry out merely a minor adjustment of the decision on the merits, but involves a total and independent re-evaluation of the non-pecuniary suffering.

The independent re-evaluation exercised by the higher courts is empirically documented in similar continental systems (civil law) by Amaral-Garcia et al. [34]. Analyzing medical malpractice litigation, the researchers highlighted that appellate judges do not have tendencies to favor medical service providers, but act as a filter of legal calibration. This pattern conceptually explains why our Wilcoxon test did not detect a unidirectional tendency of increase or reduction, while at the individual level the AAR and AR oscillations remain extreme. The appellate court intervenes on a case-by-case basis, censuring the excesses of the first instance, but also substantially increasing the compensation in the cases where the suffering caused by death was initially underestimated.

Overall, the results indicate that the process of establishing the amount of non-pecuniary compensation on appeal has a strongly individualized character, the intervention of the higher court being influenced by the particularities of each case, rather than by a logic of proportional adjustment of the amount initially established.

At the same time, the amplitude of the modifications observed raises questions concerning the degree of predictability of the quantification of non-pecuniary compensation in judicial practice. The fact that the appellate court may significantly reduce or increase the compensation established at first instance suggests the existence of differences in appreciation between the jurisdictional levels regarding the evaluation of non-pecuniary suffering.

### 4.3. Amounts Established in Similar Cases

Comparing similar cases reveals wide variation in the amount of non-pecuniary compensation awarded by the courts, even where the facts are closely alike. The results obtained suggest that the establishment of the amount of non-pecuniary damages depends to a considerable extent on the individual appreciation of the court in each case.

In the case of the death of newborns, although the harm and the capacity of the main civil parties were similar, the amounts awarded varied substantially at first instance from €5000 up to 50 times more per party. The appellate court harmonized the non-pecuniary damages of the persons having the capacity of parents of the deceased, awarding €25,000, €35,000, and €40,000 respectively. However, in one of the cases there were differences in the sense that the mother was awarded an amount higher by €10,000 than the father of the deceased newborn. The observed variations indicate that, even within comparable circumstances, the courts may evaluate differently the intensity of the non-pecuniary harm and the level of compensation considered equitable.

A very significant variation was observed in the cases in which the civil parties were minor children left without one of the parents. In the two analyzed cases, the amount of compensation awarded to the minor child varied between €5000 and €100,000, a 20-fold difference between the damages established in the two situations. This discrepancy is even more relevant given that the age of the children and the general circumstances of the harm presented comparable elements.

Similarly, in the cases concerning the death of a wife and mother, important differences are observed both in the total amount of compensation awarded (€150,000) and in its distribution among the civil parties (€125,000 difference in the capacity of husband from one case to another). In the case in which there were several civil parties, the courts awarded differentiated compensation among husband, child, and parents, and on appeal their amount was significantly increased by between 67% and 141%. The comparison of these cases highlights that the evaluation of non-pecuniary harm may vary according to the way in which the court appreciates the family relationships and the impact of the death on each harmed person.

The diversity of amounts in cases with similar features finds a fundamental explanation in the reference study carried out by Diamond et al. [35]. Analyzing the internal dynamics of court trials, the researchers demonstrated that, in the absence of objective benchmarks for calculation, the evaluation of human suffering undergoes a structural unpredictability, being strongly influenced by the variability of the individual perceptions of those who judge. This conclusion directly explains the major discrepancies identified in our sample, suggesting that jurisprudential variability is a direct consequence of the lack of standardized guidance criteria. Explaining why the amounts diverge between otherwise comparable cases requires interpreting the reasons the courts give; this lies beyond the quantitative scope of the present pilot study and is the object of a separate one.

At the same time, the differences observed between the amounts awarded in comparable cases may raise problems of predictability of judicial practice. In the absence of guiding benchmarks concerning the evaluation of non-pecuniary harm, the appreciation of the courts may lead to contrasting solutions in similar situations, which may influence the perception of the coherence of the act of justice.

This vulnerability is not, however, isolated; comparative studies carried out in similar continental systems [36,37] show that throughout the tradition of European civil law, the absence of mandatory scales or orientation guidelines inevitably leads to an asymmetric and inequitable distribution of compensation. The data from our sample are consistent with these international models, indicating that the lack of national benchmarks in the matter of medical malpractice leaves the quantification of non-pecuniary damages vulnerable to judicial subjectivism, directly affecting the predictability of the act of justice in Romania.

### 4.4. Impact on Healthcare Professionals

Beyond their strictly legal significance, the patterns of unpredictability documented above bear directly on the professionals exposed to this form of litigation. A physician’s exposure to a malpractice accusation is recognized in the specialized literature as a traumatic event in itself, whether as the Medical Malpractice Stress Syndrome (MMSS), established by Ryll, or the Clinical Judicial Syndrome described by Gómez-Durán; both represent a profound bio-psycho-social imbalance of the individual under the pressure of the litigation/trial [25,26,27,28]. From a clinical standpoint, the two entities share a common core of somatic and emotional manifestations consisting of sleep disturbances, anxiety, panic, depressive states, chronic fatigue, or the exacerbation of pre-existing conditions. On the behavioral and social level, both syndromes broadly translate into isolation and alienation, with the individual displaying deep feelings of frustration, indignation, and defensiveness, which often lead to the deterioration of family and professional relationships.

This clinical picture is fed directly by what is termed “critogenic harm”—that is, an inherent and unavoidable psychological injury inflicted on medical personnel by the aggressiveness and hostility of the legal apparatus [25]. In this traumatic context, the profound fear of losing one’s personal integrity and the uncertainty regarding the outcome of the litigation [38] become the principal vectors that sustain the stress symptoms throughout the legal proceedings. In the Romanian setting, the absence of predictable judicial standards is likely to aggravate this critogenic harm as professionals are unable to anticipate the magnitude of their potential liability and therefore face an additional, structural source of uncertainty that compounds the psychological burden of the proceedings.

Extending this discussion, Vizcaíno-Rakosnik et al. demonstrate in a recent study that specific exposure to criminal proceedings raises the rate of clinicians who develop a notable adverse psychological reaction to 67.53% (*p* = 0.0206) [39]. Consequently, the mere involvement in judicial proceedings and the confrontation with the prospect of criminal liability trigger the trauma, which is clinically expressed through a phenomenon of chronic rumination and systematic re-experiencing of the negative events related to the accusation, reported by more than half of the practitioners (53.5%) [39].

In line with the international dynamic, the reality of the domestic medical context is faithfully reflected by a qualitative study conducted in Romania, which analyzed the impact of liability complaints, whether civil, disciplinary, or criminal, on the medical profession. Its results showed that Romanian physicians exposed to investigative procedures are profoundly affected, reporting at the personal level insomnia, nightmares, stress, and anxiety, and at the professional level a decline in confidence in their own clinical decisions, fear, and tendencies toward isolation. Moreover, the study documented that this climate of uncertainty and legal pressure forces Romanian practitioners to adopt defensive medicine strategies as a protective mechanism, which materialize either through additional practices (ordering unnecessary investigations and interclinical consultations) or through avoidance practices (e.g., declining complex cases) [40].

Furthermore, it is well established that this stress has a direct impact on the well-being of healthcare professionals and on the way they choose to practice their profession, generating defensive medicine practices and a reduction in the time spent with patients [37,41]. In support of Gómez-Durán’s conclusions, the research by Arimany et al. is also relevant, as it reported that the professional’s well-being is important in determining the quality of the care they provide [42].

By deduction, if the simple initiation of proceedings through the filing of a complaint can have such significant effects on the clinician’s psychological balance, then the unpredictability we have documented could plausibly amplify this occupational stress—a possibility grounded in the existing literature. Whether it actually does so is a question for future research rather than a finding of the present study. The awareness that the management of professional risk is subject to a structural unpredictability—in which the amount of moral damages does not follow a proportional logic (the slope of our regression being extremely low, and the FI values not constituting a predictor of AC)—deprives the professional of any benchmark of equity and predictability regarding their patrimonial risk within a medical malpractice trial.

Beyond the psychological mechanisms of the anchoring effect, this form of dependence raises a serious ethical problem in the process of assessing medical liability. The fact that our study suggests that almost 60% of the variation in the amounts awarded may be determined by the level of the claims requested reveals a possible distortion of the concept of judicial equity. In an ideal legal system, the amount of compensation for non-pecuniary harm should reflect the gravity of the medical fault, the clinical consequences, and the intensity of the human suffering generated by the death.

The econometric reality identified in our sample indicates that the final value of the compensation imposed on healthcare professionals may be influenced, to some extent, by the aggressiveness or financial strategy of the representatives of the civil parties. The asymmetry highlighted transforms medical liability from a compensation mechanism into a factor that destabilizes professional autonomy and a source of stress for practitioners (who realize that their financial protection depends not on adherence to clinical guidelines but, to a certain extent, on the amount of the claims in the file).

This patrimonial exposure is, moreover, only partially mitigated by insurance. Romanian law makes professional liability (malpractice) insurance mandatory for every physician and nurse, as well as for medical institutions [4]. For individual practitioners the minimum insured limits set by law range from €4000 to €62,000, depending on the category of personnel (from nurses to physicians) and, for physicians, on whether the specialty is clinical, paraclinical or surgical; for hospitals they range from €100,000 to €500,000 depending on the hospital’s grade [43]. Although these are legal minimums and may therefore be increased, in practice they are frequently treated as fixed, and, depending on the policy, insurance typically caps the payout for moral damages at a small and variable proportion (commonly 10–25%) of the total insured sum [44,45]. Consequently, upon conviction, insurance frequently does not cover the full compensation established by the court, and this shortfall concerns above all the moral damages. Contrary to the intuition that liability insurance would neutralize the financial exposure, the combination of low, often-fixed limits and only partial coverage of moral damages means that a substantial and unpredictable portion of the award may fall directly on the professional; the unpredictability we document may thus add to the financial and psychological pressure on the professional.

### 4.5. Strengths and Limitations

To the best of our knowledge, this is the first study in Romania to address the quantification of non-pecuniary damages in the context of criminal medical liability and negligence. Thus, the present research opens a new direction of research in the jurisprudential evaluation of medical malpractice, transforming a field previously ignored econometrically into a benchmark of analysis.

At the same time, the innovative character of the present study resides in its methodological approach. The research introduces an original model of econometric analysis of non-pecuniary damages, structured on proportional indicators (CTAR, CRR, AAR, AR) capable of extracting and standardizing the patterns of judicial decision, alongside applied statistical analysis. The main strength of this methodological framework is its scalability and universality; the model can thus be replicated directly on extended sets of rulings belonging to other European or international legal systems, allowing the carrying out of cross-border comparative studies. In this respect, civil law systems such as Romania’s grant judges broad individual discretion in quantifying non-pecuniary damages, in contrast to common law jurisdictions, which makes the proposed framework particularly transferable to other civil law countries. Through the methodology followed, our study highlights the structural differences in the quantification mechanisms and measures, in an objective manner, and the degree of predictability and equity in the matter of medical liability. Future directions of research should aim at extending the database nationally. Beyond national extension, future work should apply the framework to non-fatal negligence, correlate the documented jurisprudential unpredictability with validated occupational stress instruments such as the Physician Work Life Scale, and incorporate studies examining judicial reasoning underlying the quantification of damages.

The main limitation of the present study derives from the small size of the sample (*n* = 10). For this reason, our results have a pronounced exploratory character and cannot be generalized to the level of the entire Romanian judicial practice. In particular, the first-instance-to-appeal analysis rests on only six appealed cases, so its statistical power is very limited.

A further limitation is that this study did not directly measure mental health or well-being outcomes: it documents an objective, quantifiable stressor—the structural unpredictability of moral damages—whose psychological impact on professionals is inferred from the existing literature rather than assessed empirically. Future research should correlate this jurisprudential unpredictability with validated indicators of occupational stress and well-being.

## 5. Conclusions

The level of non-pecuniary damages awarded in the studied cases indicates the absence of a recognizable pattern of quantification, despite the fact that the analyzed cases concern the same criminal offense and the same nature of the harm, namely the death of a patient resulting from medical negligence. In this context, the variability of the amounts, reflecting a lack of uniformity in how the magistrates understand to rule on non-pecuniary damages, may affect perception of the coherence of judicial practice. In the field of professional medical liability, where the legal consequences intersect with profound social and emotional implications, the predictability of judicial practice becomes an important element for both harmed persons and healthcare professionals.

On the basis of an empirical analysis, the results of the present study highlight the amplitude of the variation existing in the non-pecuniary damages awarded by the courts in Romania. Identifying this variability represents a first step toward exploring instruments that could contribute to increasing the coherence and transparency of the process of quantifying non-pecuniary damages in cases of patient’s death associated with the medical act. As a pilot study, the present work is exploratory, so its purpose is to establish that the proposed method is reproducible and scalable before it is applied to larger datasets, rather than to provide a definitive account of Romanian judicial practice.

From this perspective, the results of the research offer medical personnel a pragmatic basis for awareness and management of the legal risk of practicing the profession. Given the extreme variability of the amounts, the study demonstrates the magnitude of the unpredictability that physicians and nurses face before the Romanian criminal courts. Thus, the analysis directly benefits the medical community by providing objective perspectives that raise concerns about the consistency of judicial practice and suggests that further investigation is warranted for reducing physicians’ exposure to an otherwise unpredictable patrimonial risk in the process of adjudicating the medical act. Ultimately, reducing this structural unpredictability should be regarded not merely as a matter of judicial coherence but also as a measure capable of protecting the occupational well-being of physicians and nurses, mitigating a litigation-related stressor that, as our findings suggest, currently lies largely beyond their control.

The authors acknowledge that no two cases are identical and that a rigid schedule imposing an identical sum on every case would be neither feasible nor desirable. Future research should therefore examine whether, and to what extent, greater consistency in the quantification of non-pecuniary damages could be achieved without limiting the courts’ ability to individualize each award. In this regard, existing instruments used in other areas of personal injury compensation—including the Baremo in Spain, the Ogden Tables in the United Kingdom, the Mornet framework in France, and the guidance developed for compulsory motor third-party liability insurance (RCA) in Romania—may provide useful comparative points of reference for further investigation. Building on the present pilot analysis, our aim in future work is to assess whether elements of such approaches could inform the development of more consistent and transparent methods for evaluating non-pecuniary damages in medical malpractice cases resulting in a patient’s death.

## Figures and Tables

**Table 1:** Comparative presentation of non-pecuniary damages in the analyzed rulings. ^1^ The amounts presented were, as the case may be, claimed/awarded per civil party unless otherwise specified. ^2^ In this case, the appellate court removed a civil party from the proceedings. ^3^ In the ruling pronounced by the appellate court, the separate opinion of a judge was noted, who considered it equitable and proportionate to award non-pecuniary damages of €87,000/party. ^4^ The appeal against the first-instance ruling was either not lodged or not admitted, so that the ruling already pronounced remains final.

Case No.	Capacity of the Entitled Person	Amount of Non-Pecuniary Damages Claimed ^1^	Non-Pecuniary Damages Awarded by the First-Instance Court ^1^	Non-Pecuniary Damages Awarded by the Appellate Court ^1^	Total Value of Non-Pecuniary Compensation per Case
C1	Father, sister, brother	€50,000	€15,000	€20,000	€40,000 ^2^
C2	Mother, father	€500,000	€150,000	€40,000	€80,000
C3	Mother, father	€1,500,000	€250,000	€40,000 ^3^	€80,000
C4	Wife, son, daughter	€500,000	€40,000	- ^4^	€120,000
C5	Wife, son	€500,000	€100,000	- ^4^	€200,000
C6	Wife, son	€500,000	€100,000 wife €50,000 son	€40,000 wife €25,000 son	€65,000
C7	Husband	€900,000	€50,000	€75,000	€75,000
C8	Husband, son, mother, father	€1,000,000	€83,000 husband €80,000 son €30,000 mother €30,000 father	€200,000 husband €100,000 son €50,000 mother €50,000 father	€400,000
C9	Daughter, mother, father, two sisters	€6000 daughter €4000 mother, father and sisters	€5000 daughter €3000 mother, father and sisters	- ^4^	€17,000
C10	Mother, father, two brothers	€200,000 mother, father €100,000 brothers	€35,000 mother €25,000 father €5000 brothers	- ^4^	€70,000

**Table 2:** Comparative presentation of non-pecuniary damages across both courts. ^1^ The result is marked with “+” if AR > 1, respectively, if AC increased the non-pecuniary damages established by FI. Likewise, the result is marked with “−” if AR < 1, signifying that AC reduced the damages pronounced by FI.

Case No.	FI	AC	AR	Result ^1^
C1	€30,000	€40,000	1.33	+
C2	€300,000	€80,000	0.27	−
C3	€500,000	€80,000	0.16	−
C6	€150,000	€65,000	0.43	−
C7	€50,000	€75,000	1.50	+
C8	€223,000	€400,000	1.79	+

**Table 3:** Comparative presentation of non-pecuniary damages in the analyzed rulings. ^1^ The appeal against the first-instance ruling was either not lodged or not admitted, so that the ruling already pronounced remains final.

Case No.	The Deceased	Capacity of the Civil Party	Non-Pecuniary Damages Claimed	Damages Awarded at First Instance	Damages Awarded at Appeal
C2	Newborn	Mother, father	€500,000/party	€150,000/party	€40,000/party
C3	Newborn	Mother, father	€1,500,000/party	€250,000/party	€40,000/party
C10	Newborn	Mother, father, brother	€200,000 mother, €200,000 father, €100,000 brother	€35,000 mother €25,000 father €5000 brother	- ^1^

**Table 4:** Comparison of similar cases concerning the death of a wife/mother.

Case No.	The Deceased	Capacity of the Civil Party	Non-Pecuniary Damages Claimed	Damages Awarded at First Instance	Damages Awarded at Appeal
C7	Wife	Husband	€900,000/party	€50,000 husband	€75,000 husband
C8	Wife	Husband, son, mother, father	€1,000,000/party	€83,000 husband, €80,000 son, €30,000 mother, €30,000 father	€200,000 husband, €100,000 son, €50,000 mother, €50,000 father

**Table 5:** Comparison of similar cases concerning the death of a parent with a minor child. ^1^ The appeal against the first-instance ruling was either not lodged or not admitted, so that the ruling already pronounced remains final.

Case no.	The Deceased	Capacity of the Civil Party	Damages Awarded at First Instance	Damages Awarded at Appeal
C8	Mother	Minor son	€80,000	€100,000
C10	Father	Minor daughter	€5000	- ^1^

## Data Availability

The data analyzed in this study are contained within the article (Table 1, Table 2, Table 3, Table 4 and Table 5). They were extracted from final court rulings that are publicly available, in anonymized form, through the rejust.ro portal of the Superior Council of Magistracy of Romania (https://rejust.ro). Further inquiries can be directed to the corresponding author.
